# The effect of Myo-inositol on fertility rates in poor ovarian responder in women undergoing assisted reproductive technique: a randomized clinical trial

**DOI:** 10.1186/s12958-021-00741-0

**Published:** 2021-04-23

**Authors:** Sahar Mohammadi, Fatemeh Eini, Fatemeh Bazarganipour, Seyed Abdolvahab Taghavi, Maryam Azizi Kutenaee

**Affiliations:** 1 Fertility and Infertility Research Center, Hormozgan University of Medical Sciences, Bandar Abbas, Iran; 2 Social Determinants of Health Research Center, Yasuj University of Medical Sciences, Yasuj, Iran

**Keywords:** IVF, Myo-inositol, Ovarian sensitivity index, Poor-responder

## Abstract

**Background:**

Poor ovarian response to gonadotropin is a significant challenge in assisted reproductive technique (ART) and affect 9–24% of ART cycles. This study aimed to evaluate the effect of Myo-inositol on fertility rates in poor ovarian responder women undergoing assisted reproductive technique.

**Methods:**

This study is a double-blinded randomized controlled study that involved 60 poor ovarian responders included in an ICSI program and divided into two groups; intervention group: 30 patients who have been assuming Inofolic (4 g myo-inositol + 400 μg folic acid) for the before the enrollment day; control group: 30 patients assuming folic acid (400 μg) for the same period. Controlled ovarian stimulation was performed in the same manner in the two groups. The main outcomeswere the assessment of oocytes retrievednumber and quality, ovarian sensitivity index,required dose of Gonadotropinsunits × 1000), fertilization rate, biochemical, and clinical pregnancy rate.

**Result:**

There is no significant difference in clinical characteristics between study groups. The number of oocytes retrieved, number of MII oocytes, number of embryos transferred, chemical, and clinical pregnancy were higher in the intervention group. However, they are not statistically significant in comparison to the control group. The ovarian sensitivity index and fertilization rate were significantly higher in the intervention group than the control group (*P* > 0.05). The required dose of gonadotropin significantly lower in the intervention group than the control group.

**Conclusion:**

Our results suggest that the supplementation myo-inositol in poor ovarian responders significantly improved the ART outcomes such as fertilization rate gonadotropin, ovarian sensitivity index (OSI) and significantly reduced the required unities of gonadotropin. Additionally, more extensive randomized controlled studies are needed.

**Trial registration:**

Iranian Registry of Clinical Trials, IRCT20180515039668N1, retrospectively registered since 2020-03-16.

## Background

The poor ovarian response is the most important limiting factors in the success of in-vitro fertilization (IVF), which is observed in 9–24% of women which are undergoing assisted reproduction techniques [1, 2]. In people with poor ovarian response can be seen ovulation reduction, decreased fertility following treatments due to increased age or reduced ovarian reserve [3]. Ovarian reserve as a potential marker of ovarian function is to reflect the quantity and quality of the eggs within an ovary [4]. Fertility outcomes directly depend on the quality of the egg; in fact, low-quality eggs may cause female infertility and an important obstacle in in vitro fertilization [5].

The quality of the eggs depends on the various processes that occur during the oogenesis, and the oocyte growth and maturation depend on the follicular microenvironment [6]. Some biochemical characteristics of follicular fluid surrounding oocyte may play a decisive role in determining the quality of the egg, and they arepotential factors for achieving fertilization and fetal development [6, 7]. Recent studies have also shown that high concentrations of myo-inositol in follicular fluid have a significant role in follicle maturation and markers of good quality ovulation [8].

Myo-inositol is an enzymatic isoform of inositol and belongs to the vitamin B complex family [9]. It is produced from glucose 1-phosphate in a NAD-catalyzed oxidation reaction with nicotinamide adenine dinucleotide (NAD). Myo-inositol is cyclic alcohol that plays a crucial role in cell morphogenesis and cytogenesis, signal transduction, lipid synthesis, cell membrane structure [10]. In the ovary, Myo-inositol is responsible for important intracellular signals essential for oocyte development, because it seems to improve oocytes in vitro maturation. Moreover, Myo-inositol induces the intracellular pathways are involved in the release of cortical granules, in the inhibition of polyspermy, in the completion of meiosis and in the activation of the cell cycle that subsequently results in embryo development [11] and reduce the oxidative stress [12]. Thefindings of various studies have shown that administration of myo-inositol in patients with polycystic ovary syndrome (PCOS) undergoing assisted reproductive techniqueshas positive effects on insulin sensitivity, oocyte maturation, egg quality, and pregnancy rate. That is why the administration of inositol is one of the treatments of polycystic ovary syndrome [13]. Moreover, Caprio and colleagues [14], in a pilot study, have reported that treatmentwith myo-inositol could have a positive effect on the fertilization rate, implantation rate and pregnancy rate in poor ovarianresponders.

As previously mentioned,patients with poor ovarian response havedeclined in the number of oocytes andthehighimplantation failure rates. Since fertility outcomes depend directly on the quality of the oocytes,we designed a randomized clinical trial that aimed to evaluatethe effect of myo-inositol on fertility rates in poor ovarian responder women undergoing assisted reproductive technique.

## Methods

### Design and data collection

This study is A randomized controlled clinical trial with double-blinded parallel groupsregistered at theIranian Registry of Clinical Trials, IRCT20180515039668N1, retrospectively registered since 2020-03-16. Also, this study approved by Hormozgan University of Medical Sciences Ethics Committee with ethical code IR.HUMS.REC.1398.393. Sixty infertile women with poor ovarian response attending at the infertility centerof Hormozgan University of Medical Sciences in Bandar Abbas, Hormozgan, Iran, were invited to participate in the study during 2020. An information sheet was offered to all women and informed written consent was obtained. All participants were aware that they couldwithdraw any time during the trial. Randomization was centralized and computerized with a concealed randomization sequence. According to the study of Lesoine et al. [15] and α = 0.05, β =0.2, the sample size were estimated at least 30 women per group.

### Recruitment of participants

The inclusion criteria were infertile women of 20–43 years old who have one of the criteria of poor ovarian responder as bellow: Antral follicle count less than 7 orAnti-Mullerian hormone level Less than 1.2 ng/ml. Other criteria were Having a body mass index (BMI) of 19–25, absence of endocrine and metabolic disorders such as polycystic ovary syndrome, hyperprolactinemia, diabetes and thyroid dysfunction Pelvic pathology such as hydrosalpinx, uterine anomaly Stages III to IV endometriosis and fibroma, absence ofmale factors infertility such as Oligo-Astheno-Teratozoospermia (OAT) or Azoospermia. The study excluded patients who become pregnant spontaneously in pretreatment period and they have no desire for cooperation.

### Study design

All patients will receive infertility treatment with the antagonist protocol after receiving the drug. After ovulation induction, the number of oocytes, fertilization rate, and pregnancy rate are checked. Blinding: People in the control group receive folic acid powder similar to the form of Inofolic (myo-inositol + folic acid) produced by a reputable private pharmaceutical company. Both groups of patients, clinical researcher, and data analyzer, do not know the type of treatment.

All patients were divided into two pretreatment groups: Intervention group who receive Infolicpowder 4 g/daily-12 weeks and Control group who receive folic acid 400 mg/ daily-12 weeks. In the last cycle of pretreatment, two ampoulespergoveris (Merck-Serono, Switzerland) was prescribed daily from the second day of menstruation for 5 days. Then vaginalultrasonography using a 6–9 MHz convex-array transducer (Ultrasonix RP, Vancouver, BC, Canada) was done, and the GnRH antagonist (Cetrotide 0.25 mg sc, Merck-Serono, Switzerland) was prescribed daily. It was continued until the day of ovulatory hCG administration according to the ovarian response. The patientswere evaluated during 12–16 days of menstrual cyclesfor the response of the drug, the size of the follicles reachedin the ovary, and the thickness of the endometrium by vaginalultrasonography using a 6–9 MHz convex-array transducer (Ultrasonix RP, Vancouver, BC, Canada). When at least twofollicles were greater than 18 mm, 10,000 IU urinary hCG (Choriomon, IBSA, Lugano, Switzerland) Intramuscularly was administered for ovulation induction, and oocyte pickup was performed 34–36 h later.

The main outcomes were oocyte quality, oocytes number, Fertilization rate, Embryo quality, Clinical, Biochemical pregnancy rate, andabortion rate.

Biochemical pregnancy was defined as a small and transitory increase in β-hCG levels. The clinical pregnancies were identified by the presence of a gestational sac on ultrasonography 5 weeks after hCG injection.

### ICSI procedure

Oocyte and sperm prepared for conventional intracytoplasmic sperm injection (ICSI). Briefly, after oocyte retrieval, cumulus and corona radiata cellswere immediately removed after retrieval by a short exposure to Life global total HEPES (Life global, Brussels, Belgium) containing 20 IU/mL hyaluronidase (Sage IVF) and gentle aspiration in and out ofa Pasteur pipette and mechanically cleaned from the remaining surrounding cumulus cells by aspiration using a denuding pipette (Denuding Flexi-Pet; Cook, Brisbane, Australia) with 130–170 mm diameter. The denuded oocytes were assessedfor their meiotic maturation status. The oocytes with an extruded first polar body presumably atthe metaphase II stage (MII) were selected for ICSI. Semen was prepared by discontinues density gradient based on factory instruction. We make the lower (90%) phase and upper (45%) phase gradient by AllGrad 100 and AllGrad wash solution (Life global, Brussels, Belgium). Liquefied semen gently placed onto the upper phase and Centrifuged for 18 min at 350 g. ICSI procedure has been described previously [16] and performed at 4–6 h after oocyte retrieval. After ICSI, the resulting embryos were classified base on quality, and good quality embryos were transferred 3 days after ICSI.

#### Statistical analysis

Descriptive and analytical statistical using SPSS 21 (SPSS, Chicago, IL, USA) was used in the present study. Data is presented as average (standard division) for quantitative variables and number (percent) for qualitative. In order to compare groups, we used unpaired t-test for quantitative variables and × 2 test for ordinal variables. A *p*-value less than 0.05 was considered statistically significant.

## Result

### Sample characteristics

**Fig. 1:**
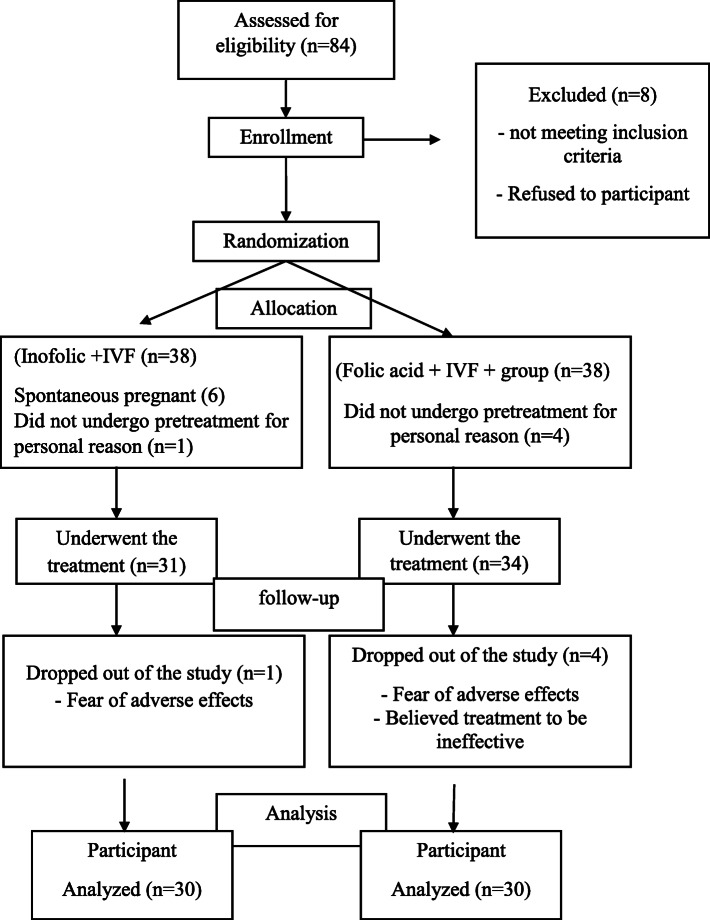
The process of allocating participant in 2020

Seventy-six eligible patients randomly divided into two groups (38 subjects in each group). Among recruited patients, five declined to participate for personal reasons, four in control (folic acid), one in intervention group (myo-inositol + folic acid), as well as six patients excluded due to spontaneous pregnancy during pretreatment in intervention group. The patients who continued the study were 65.31 subjects were placed in intervention group, 34 in control group. Finally, one patient in intervention (folic acid), four patients in the control group were not continued the study. The process of allocating participants in2020 is shown in Fig. 1.

Clinical characteristic of the patients is presented in Table 1. There is no significant difference in appearance between study groups (*P* > 0.05) (Table 1).

**Table 1:** Clinical characteristics of the patient in study groups. The data show that all groups were comparable (*P* ≤ 0.05)

Variable	Intervention group (*n* = 30)	Control group (*n* = 30)	*P* value
Age (year)	35 ± 6.91	36.7 ± 5.6	0.46
AMH	0.92 ± 0.68	1.15 ± 0.59	0.16
AFC	6.80 ± 3.08	7.93 ± 2.8	0.14
Follicle (n)	3.68 ± 3.02	2.76 ± 1.53	0.14
FSH	7.89 ± 2.91	6.72 ± 3.06	0.15
Estradiol	2012.92 ± 4.94	1861.19 ± 1.62	0.75

Table 2 shows the outcomes of assisted reproductive technique in both groups of the poor ovarian responder. The number of oocytes retrieved, number of MII oocytes, number of embryos transferred, chemical, and clinical pregnancy were higher in the intervention group. However, they are not statistically significant in comparison to the control group (*P* > 0.05). Whereas the ovarian sensitivity index (OSI)) (calculated by dividing the total administered rFSH dose by the number of oocytes retrieved to obtain the FSH-to-retrieved oocyte ratio [17]) and fertilization rate were significantly higher in the intervention group than the control group (*P* > 0.05). Moreover, the miss abortion rate in the intervention group was higher than the control group, but it was not significant (*P* > 0.05). Statistical analysis has shown that the required dose of gonadotropin significantly lower in the intervention group than the control group.

**Table 2:** The comparison of the main outcomes in study groups. The data show that there is no significant difference between all groups (*P* ≤ 0.05)

Variable	Intervention group (*n* = 30)	Control group (*n* = 30)	*P* value
Gonadotropin 450	4752 ± 432	5490 ± 492	0.000
Gonadotropin dose	10.56 ± 0.96	12 ± 1.09	0.000
Ovarian stimulation index	74 ± 0.7	0.43 ± 0.27	0.04
No. of Oocyte	3.4 ± 3.11	2.3 ± 1.28	0.11
Oocyte retrieved (n)	3.4 ± 3.11	2.3 ± 1.28	0.11
MII oocyte (n)	2.36 ± 1.64	1.87 ± 1.07	0.24
Fertilization rate (%)	68.6 ± 35.4	40.75 ± 39.44	0.02
Embryo transferred (n)	1.89 ± 1.1	1.20 ± 0.83	0.29
Chemical pregnancy	6 (20%)	3 (10%)	0.3
Clinical pregnancy	2 (6.6%)	0	0.15
Miss abortion	4 (13.3%)	3 (10%)	0.31

## Discussion

To our knowledge, for the first time, we designed a randomized clinical trial that evaluates the effect of myo-inositol on the outcome of the IVF cycle in patients defined as the poor ovarian responder. Our data have shown that myo-inositol therapy significantly decreases the required dose of gonadotropin in poor ovarian responders, and also it significantly increased the ovarian sensitivity index and fertilization rate in poor ovarian responders. Moreover, the number of retrieved oocytes, the number of MII oocytes, the number of embryos transferred, chemical and clinical pregnancy rate showed a positive trend in patients treated by myo-inositol without reaching a statistical significance.

Our data were in line with the result of a controlled observational study of Francesca Caprio, and collogues [14] that reported the myo-inositol therapy was associated with an enhancement in the fertilization rate, implantation rate, grade I embryos rate and pregnancy rate, but they were no statistically significance. Moreover, they have reported that the number of retrieved MII oocyte and ovarian sensitivity index to gonadotropins were significantly higher in poor ovarian responders treated by myo-inositol. As well as, Lisi and colleagues [18] evaluated the effect of myo-inositol combine with folic acid on the quality of oocyte and ovarian response in women without PCOS underwent ART. In consistent to our study, the authors have reported that although the duration of stimulation was similar in myo-inositol and control groups, the total amount of gonadotropin used for follicular maturation was significantly lower in myo-inositol group. Moreover, there was no significant difference between the two groups in the implantation and pregnancy rates. Recently it has been proposed that the ovarian reserve markers including patient’s age, serum day-three FSH, and AMH level showed a good effectiveness for the choice of FSH starting dose and for optimizing the controlled ovarian stimulation during IVF [19]. Therefore, it seems that determination of gonadotropin stating dose in addition to myo-inositol may reduce the required gonadotropin particularly in poor ovarian responder. The possible reduction of the required gonadotropin, as well as the duration of controlled ovarian stimulation may significantly reduce the costs of the IVF due lower number of gonadotropin vials and reduced number of outpatient accesses for the follow-up.

Even the other studies which evaluate the effect of inositol on fertility outcomes in other diseases such as PCOS, their findings were resembled to our finding. Since, accumulating evidence has shown that one of the most important mechanisms of PCOS pathogenesis is the insulin-resistance. For this reason, the use of insulin-sensitizers, such as inositol isoforms, gained increasing attention due to their safety profile and effectiveness. It is noteworthy that there are a specific myo-inositol depletion and D- chiro-inositol overload in the ovary of PCOS patients [20], therefore the restoring of the physiological level of these isomers in the follicular fluid could be correct the ovarian function [21]. For example, Pourghasem et al. [22] compared the effectiveness of myo-inositol and metformin in infertile women with polycystic ovary syndrome treated with letrozole. The authors reported that the addition of inositol and metformin to the treatment of infertile PCOS women with letrozole resistance improves the ovarian function; however, it is not significant. Of note, inositol was more effective than metformin in patients. As well as, Bernd Lesoine and Pedro-Antonio Regidor [15] investigate the effect of myo-inositol with folic acid on oocyte quality, the ratio between follicles and retrieved oocytes, the fertilization rate, and the embryo quality in PCOS patients undergoing ART treatments. Their findings have shown that myo-inositol therapy in women with PCOS results in better fertilization rates and a clear trend to better embryo quality as the number of retrieved oocytes was smaller in the myo-inositol group.

Previous studies have shown that the myo-inositol as an important component of follicular fluid, playing a crucial role in both nuclear and cytoplasmic oocyte development [11, 23]. Moreover, research has shown that the administration of myo-inositol can result in calcium release through the interaction of myo-inositol and its receptors in oocytes [24]. Calcium oscillation has a pivotal role in meiotic resumption and is responsible for the final oocyte development. Previous studies support this claim that myo-inositol, as a second messenger of calcium signaling, plays a critical role in oocyte development [25]. Thus, it can be concluded that myo-inositol can improve the pregnancy rate by supporting oocyte development and modulating hormonal balance.

A high level of D-chiro-inositol has been reported to affect oocyte and blastocyst quality as well as ovarian functionality [26]. A recent study indicated that a high concentration of D-chiro-inositol could inhibit aromatase, an enzyme that converts androgens into estrogens and is involved actively in the biosynthesis of estrogens. Therefore, the altered functioning of the aromatase enzyme accounts for the impairment of androgen conversion into estrogens [27]. This adverse condition may be improved via myo-inositol supplementation, which can enhance the reproductive activities in PCOS patients. Moreover, It has been shown that serum myo-inositol could act as a trophic factor for promotion of in vitro development of preimplantation embryos [28].

Moreover, an interesting and unexpected finding in our study was that six patients excluded from the study due to spontaneous pregnancy in the pretreatment period of intervention group. This finding reflects the positive effects of myo-inositol on the fertility of poor ovarian responders, and suggests that these patients also can achieve optimal results by myo-inositol without the use of expensive IVF cycle. This finding can be explained as follow at ovarian level, myo-inositol has a crucial role in FSH signaling, and therefore, this could lead to reduction in the required dose of gonadotropin [11, 23]. Another mechanism has been proposed for the effect of inositol on IVF outcome is due to inositol potential to reduce the oxidative stress caused by different agents through the induction of natural antioxidant defenses by increasing superoxide dismutaseand catalase levels and intracellular content of glutathione [12].

Several systematic reviews and meta-analyses have shown that myo-inositol outperforms placebo in terms of ovulation and pregnancy rates. In the systematic review and meta-analysis, Pundir et al. revealed that myo-inositol, in comparison with placebo, appeared to improve the ovulation rate, and metabolic and hormonal profiles significantly in women with PCOS [29]. Furthermore, the findings of a comprehensive review by Kamenov et al. and Gateva et al. showed that the use of myo-inositol was an important therapeutic approach to improve the metabolic and reproductive disorders in PCOS patients. In the last two studies mentioned above, remarkable clinical outcomes were obtained through myo-inositol pretreatment, followed by ART protocols [30, 31].

However, this randomized clinical trial study has some limitations: the sample size is too small to offer a great significanceof the result obtained.

## Conclusion

Our results suggest that the supplementation myo-inositol in ART treatment significantly improved the ART outcomes such as fertilization rate gonadotropin ovarian sensitivity index (OSI) and significantly reduced the required unities of gonadotropin. Additionally, larger randomized controlled studies are needed.

## Data Availability

The primary data for this study from the authors on direct request.

## Abbreviations

ICSI: Intracytoplasmic sperm injection
ART: Assisted reproductive technique
IVF: In vitro fertilization
PCOS: Polycystic ovarian syndrome
HCG: Human chorionic gonadotropin
